# A Case of Autosomal Dominant Alport Syndrome Diagnosed Just Before Discontinuation of Follow-Up

**DOI:** 10.3390/pediatric18030072

**Published:** 2026-05-25

**Authors:** Yasuyo Kashiwagi, Hironobu Okuno, Takahito Moriyama, Natsuko Inagaki, Gaku Yamanaka

**Affiliations:** 1 Department of Pediatrics and Adolescent Medicine, Tokyo Medical University, 6-7-1 Nishishinjuku, Shinjuku-ku, Tokyo 160-0023, Japan; 2 Department of Nephrology, Tokyo Medical University, Tokyo 160-0023, Japan; 3 Clinical Genetics Center, Tokyo Medical University Hospital, Tokyo 160-0023, Japan

**Keywords:** Alport syndrome (AS), autosomal dominant AS (ADAS), microhematuria, genetic testing, differential diagnosis

## Abstract

Persistent microscopic hematuria in children is often considered benign, yet recent evidence shows that a substantial proportion of affected individuals have underlying glomerular disease, particularly collagen IV-related nephropathies. We report a case of autosomal dominant Alport syndrome (ADAS) diagnosed just before discontinuation of long-term follow-up in a young woman initially presumed to have benign familial hematuria. The proband had persistent microscopic hematuria from early childhood, with normal renal function and no extrarenal manifestations. Her mother also had microscopic hematuria without kidney impairment, and the absence of accessible family history reinforced the assumption of benign familial hematuria. At age 42, the mother developed sensorineural hearing loss, and around the same time, the family learned that the maternal grandfather was undergoing dialysis for end-stage renal disease of unknown etiology. These findings prompted genetic testing, which identified a heterozygous pathogenic *COL4A4* frameshift variant (c.2317_2318del; p.Arg773GlyfsTer14) in both the mother and the proband, confirming ADAS. This case illustrates the phenotypic variability of ADAS within a single family and highlights the limitations of relying solely on clinical features or incomplete family history. In contemporary practice, persistent glomerular hematuria warrants long-term follow-up and a low threshold for molecular testing of *COL4A3-COL4A5*, even in the absence of overt clinical signs. Earlier genetic evaluation would likely have enabled a timelier diagnosis in this case. This report underscores the importance of reassessing presumed benign hematuria and integrating genetic testing into the diagnostic approach for children and young adults with persistent microscopic hematuria.

## 1. Introduction

Alport syndrome (AS) is characterized by progressive renal disease, sensorineural hearing loss, and ocular abnormalities [1]. AS has three forms: X-linked AS (the most common, about 80%), autosomal recessive AS (about 15%), and autosomal dominant AS (about <5%) [2].

X-linked AS is caused by variants of *COL4A5* on chromosome X, autosomal recessive AS by homozygous or compound heterozygous rare variants of *COL4A3* or *COL4A4*, and autosomal dominant AS (ADAS) by heterozygous rare variants of *COL4A3* and/or *COL4A4* genes, all encoding type 4 collagen.

AS is frequently identified through familial hematuria. Previously, if asymptomatic hematuria was familial and renal failure was absent, benign familial hematuria, such as thin basement membrane nephropathy, was considered. Benign familial hematuria was historically regarded as a harmless condition in which invasive diagnostic procedures such as renal biopsy were considered unnecessary. However, this descriptive term is now being replaced by genetically defined diagnoses, particularly *COL4A3*- and *COL4A4*-associated disease, as these conditions are increasingly recognized to have potential for renal progression. In the present case, we consider discontinuation of follow-up once the proband reached adulthood; just before doing so, a diagnosis of ADAS was established.

## 2. Case Report

The proband, a 7-year-old girl, first presented to our department with microhematuria detected through school urinary screening. Her microhematuria had been detected through urinary screening for 3-year-old children in Japan. Urinalysis demonstrated microhematuria (20–29 red blood cells/high-power field) without kidney function impairment. Her mother similarly had microhematuria with normal renal function without prior hearing loss or ocular abnormalities. Although the mother was estranged from her parents, her family history was unremarkable for renal disease, and we confirmed the diagnosis of benign familial microhematuria. The proband developed normally with no hearing loss or ocular abnormalities and was followed up until age 20 as having benign familial hematuria. Once the proband reached 20 years of age, we considered the discontinuation of follow-up.

**Figure 1:**
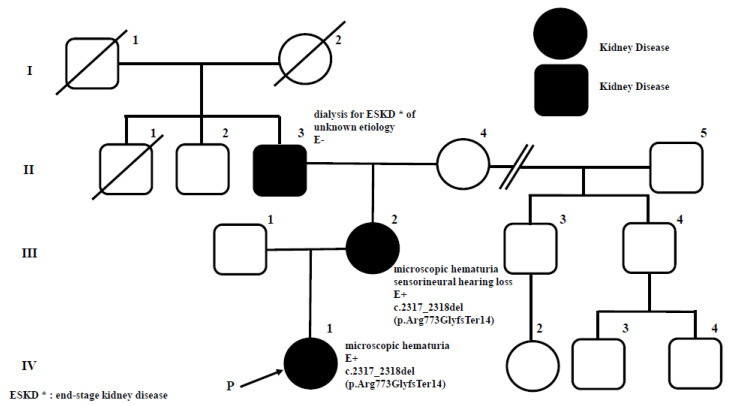
Pedigree of the family and genetic analysis of III-2 and IV-1. Circles indicate females and squares indicate males. Filled symbols represent affected individuals. A diagonal slash indicates deceased individuals. Roman numerals denote generations, and Arabic numerals denote individuals within each generation. E+ indicates microscopic hematuria; E− indicates absence of hematuria.

Around that time, her mother developed sensorineural hearing loss at 42 years old. She also reconnected with her father (the proband’s grandfather) after a long period of estrangement, and found that he was undergoing dialysis for end-stage renal failure of unknown etiology. Based on the clinical suspicion of AS, genetic testing was conducted sequentially—first in the mother and then in the proband—after obtaining informed consent. Genetic testing was performed by the Kazusa DNA Research Institute. Both were found to carry a heterozygous variant (c.2317_2318del; p.Arg773GlyfsTer14) of *COL4A4* (NM_000092.5) [Supplementary Materials]. The pedigree chart is shown in Figure 1. Because the proband’s mother had long been estranged from her parents, detailed medical histories of other family members could not be obtained. Thus, only the information shown in Figure 1 was available for constructing the pedigree.

**Figure 2:**
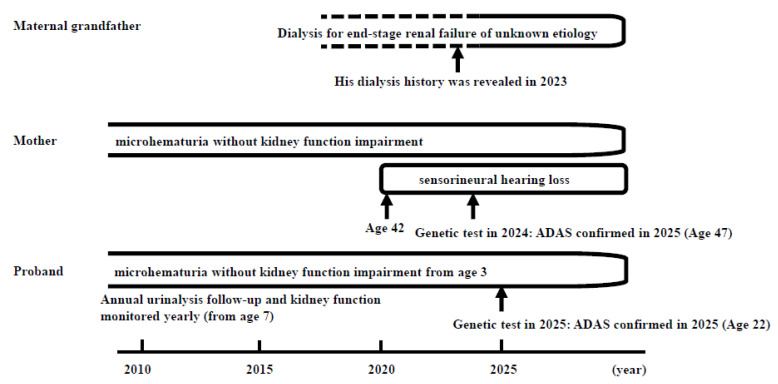
Timeline of clinical course. A dotted line indicates an uncertain or retrospectively reported period, as the exact timing of dialysis initiation was unknown.

Genetic analysis confirmed the diagnosis of ADAS in the proband and her mother. The timeline of the clinical course is demonstrated in Figure 2.

## 3. Discussion

In the present case, we confirmed the diagnosis of benign familial microhematuria and followed up until age 22 over a long period. Microhematuria is common in children and is present in 4.1% of school-age children [3]. Persistent microscopic hematuria has long been regarded as a benign condition. However, recent evidence indicates that a substantial proportion of patients actually have underlying glomerular diseases, including collagen IV-related nephropathies. In young individuals with microscopic hematuria, up to approximately 30% may miss opportunities for early kidney-protective interventions [4]. Familial microscopic hematuria represents a genetically heterogeneous group of disorders, encompassing *COL4A3/A4/A5*-associated nephropathies, heritable C3/CFHR5 nephropathy, and glomerulopathy with fibronectin deposits [5]. Notably, heterozygous *COL4A3/A4* variants, traditionally associated with thin basement membrane nephropathy (TBMN), are now recognized to predispose to progressive renal impairment, indicating that this condition is not always benign [5]. Therefore, in patients presenting with glomerular microhematuria, molecular genetic testing should be considered to enable early diagnosis and appropriate follow-up.

We summarized the differential diagnoses for persistent glomerular hematuria in this patient, including benign familial hematuria/TBMN, IgA nephropathy, and ADAS. For each condition, clinical features supporting or arguing against the diagnosis are organized in comparative Table 1. The presence of a pathogenic *COL4A4* frameshift variant, the family history of end-stage kidney disease, and the mother’s late-onset sensorineural hearing loss strongly favored ADAS over other conditions.

The concept of ADAS has evolved substantially in recent years. With the widespread availability of genetic testing, many more individuals with *COL4A3/COL4A4*-associated disease are now being identified, leading to major advances in understanding its clinical spectrum and genotype–phenotype correlations. ADAS should be considered in individuals presenting with persistent glomerular hematuria, proteinuria, or chronic kidney disease. Importantly, ADAS is sufficiently prevalent that *COL4A3/A4* variants may coexist with other renal diseases, including IgA nephropathy, complicating clinical interpretation. These recent insights indicate that ADAS is no longer a condition with “uncertain clinical, genetic, or pathological features,” but rather a well-characterized spectrum of collagen IV-related nephropathies with increasingly clear diagnostic and therapeutic implications [6].

Extrarenal manifestations of AS were also considered. Ocular abnormalities such as anterior lenticonus, perimacular flecks, and temporal retinal thinning are well-described features of X-linked and autosomal recessive AS, but are much less common in ADAS. In this family, no ocular findings were observed in either the proband or the mother. The mother developed sensorineural hearing loss in her forties, which is consistent with the reported but relatively infrequent occurrence of hearing impairment in ADAS. In Japan, a retrospective analysis of 25 patients with genetically confirmed ADAS and their family members (a total of 72 individuals from 16 unrelated families) was conducted [7], and only 1 patient was found to have hearing loss. Hearing loss in ADAS patients is a rare finding in the previous literature [7,8]. The absence of ocular involvement and the late-onset, mild extrarenal phenotype align with the known clinical spectrum of *COL4A4*-related ADAS.

The proband’s mother had been estranged from her parents for many years, and therefore, the grandfather’s history of end-stage renal disease remained unknown throughout the patient’s childhood and adolescence. This missing information contributed to the long-standing assumption of benign familial hematuria. However, in the context of current diagnostic paradigms, the delay in diagnosis cannot be attributed solely to the lack of accessible family history. Contemporary practice increasingly recognizes that persistent glomerular hematuria warrants a low threshold for molecular testing of *COL4A3***,** *COL4A4***,** and *COL4A5*, regardless of the availability of family history. Recent literature emphasizes that early genetic evaluation enables timely diagnosis and appropriate follow-up in patients with glomerular microhematuria, so genetic evaluation is the gold standard [5]. In retrospect, earlier consideration of collagen IV-related nephropathy based on the patient’s persistent hematuria alone might have shortened the diagnostic delay.

In genetic evaluation, a heterozygous frameshift variant in *COL4A4* (c.2317_2318del, p.Arg773GlyfsTer14) was identified. This variant is not listed in ClinVar and is absent from gnomAD v3.1, with an extremely low allele frequency in the Japanese population (ToMMo 0.000013). It has been reported as a pathogenic loss-of-function variant in *COL4A4* in a recent publication. According to ACMG/AMP criteria, the variant meets PVS1 (null variant in a gene where loss of function is a known mechanism), PM2 (absent from population databases), and PP4 (phenotype highly specific for *COL4A4*-related disease), and is therefore classified as Pathogenic.

The family pedigree demonstrates notable intrafamilial phenotypic variability associated with the heterozygous *COL4A4* frameshift variant. The maternal grandfather developed end-stage kidney disease requiring dialysis, whereas the proband’s mother had long-standing microscopic hematuria and developed sensorineural hearing loss in her forties, without progression to kidney failure to date. The proband presented with isolated glomerular hematuria and preserved renal function. Such variability in renal progression and extrarenal manifestations is consistent with the known phenotypic spectrum of ADAS.

In current clinical practice, earlier genetic testing could be considered in children with persistent microscopic hematuria when family history is incomplete or unreliable, even in the absence of overt clinical features. Advances in genetic testing accessibility and increased recognition of ADAS support a lower threshold for genetic evaluation in such cases. Furthermore, long-term follow-up remains essential, as clinical manifestations such as hearing loss may appear only in adulthood.

Persistent glomerular hematuria warrants long-term follow-up and a low threshold for *COL4A3–COL4A5* genetic testing, especially when family history is incomplete. Early diagnosis enables appropriate monitoring and cascade testing, and under current standards, this case would likely have been identified earlier.

## Figures and Tables

**Table 1:** Differential diagnosis in the patient. ESKD *: end-stage kidney disease.

Diseases	Supporting Features	Arguing Features
Benign familial hematuria/Thin basement membrane nephropathy (TBMN)	Persistent microscopic hematuria	Presence of ESKD * in the maternal grandfather Pathogenic *CL4A4* frameshift variant incompatible with TBMN Mother developed sensorineural hearing loss in her forties
IgA nephropathy	Microscopic hematuria	Familial clustering cannot be explained Pathogenic *COL4A4* frameshift variant identified IgA nephropathy may coexist but is unlikely to be the primary cause
X-linked Alport syndrome (XLAS)	Persistent microscopic hematuria	Pathogenic *COL4A4* variant (not *COL4A5*) Mother affected (inconsistent with classic XLAS inheritance)
Autosomal recessive Alport syndrome (ARAS)	Persistent microscopic hematuria	Only heterozygous *COL4A4* variant detected No evidence of biallelic variants Family history pattern inconsistent with recessive inheritance
Autosomal dominant Alport syndrome (ADAS)	Family history: ESKD * in maternal grandfather, microscopic hematuria in mother and proband Genetic result: pathogenic *COL4A4* frameshift variant Mother developed sensorineural hearing loss in her forties	None (clinical and genetic findings are most consistent with ADAS)

## Data Availability

The original contributions presented in this study are included in the article. Further inquiries can be directed to the corresponding author.

## Supplementary Materials

The following supporting information can be downloaded at: https://www.mdpi.com/article/10.3390/pediatric18030072/s1, Supplementary File S1: Detailed Methods and Results of the genetic analysis.
