# Supplementary material for: A Case of Autosomal Dominant Alport Syndrome Diagnosed Just Before Discontinuation of Follow-Up
Source: Pediatr Rep. 2026 May 25;18(3):72. doi: 10.3390/pediatric18030072 (PMC13304920; doi:10.3390/pediatric18030072)
Supplement: Supplementary file 1 [file pediatrrep-18-00072-s001.zip › pediatrrep-4292790-Supplementary File S1.pdf]

Methods:

Genomic DNA extracted from peripheral blood was analyzed using a targeted next-generation sequencing (NGS) panel for Alport syndrome–related genes. The coding exons and exon–intron boundaries (±10 bp) of *COL4A3*, *COL4A4*, and *COL4A5* were enriched using a hybrid-capture method and sequenced on an NGS platform (Kazusa DNA Research Institute). Sequence reads were aligned to the human reference genome GRCh38/hg38, and variants were called and annotated using standard bioinformatic pipelines. Variant frequencies were assessed using gnomAD v3.1, ToMMo 38KJPN, and ClinVar, and pathogenicity was evaluated according to the ACMG/AMP 2015 guidelines.

Results:

1) *COL4A4*: c.2317_2318del, p.Arg773GlyfsTer14

A heterozygous frameshift variant in *COL4A4* (c.2317_2318del, p.Arg773GlyfsTer14) was identified. This variant is absent from gnomAD and ToMMo databases and has been previously reported as pathogenic. According to ACMG/AMP criteria, this variant meets PVS1 (null variant in a gene where loss of function is a known mechanism) and PM2 (absent from population databases), and is therefore classified as Pathogenic.

2) Result: *COL4A5*: c.2215C>G, p.Pro739Ala

A heterozygous missense variant in *COL4A5* (c.2215C>G, p.Pro739Ala) was also detected. Although listed in HGMD, this variant is now recognized as benign, consistent with its relatively high allele frequency in population databases. ACMG/AMP classification: Benign.
